# Nursing at the London Hospital

**Published:** 1920-11-06

**Authors:** 


### Nursing at the London Hospital.

Hospital authorities in general and matrons in particular are greatly indebted to Miss Monk, R.R.C., for the able report which she has presented to the House Committee of the London Hospital on the present shortage of probationers. Prior to 1915, the hospital obtained some fourteen candidates a week, who satisfied their conditions and were admitted either into the preliminary school or direct into the hospital. The figures are worth quoting:—1910, 530; 1911, 480; 1912, 435; 1913, 486; 1914 (including V.A.D.s), 814; 1915 (including V.A.D.s), 867; 1916, 446; 1917, 354; 1918, 237; 1919 (advertising begun), 345; 1920 (to October 5), 186. Needless to say, the outlook is very serious, for advertisement in the lay papers no longer produces the results it did.

The London Hospital is meeting the situation in the wisest manner possible by improving the status of the nurses and affording better opportunities of promotion, but unfortunately these improvements do not readily penetrate to the classes from whom probationers are drawn, or are not fully realised. Considerable re-organisation has taken place. The matron will delegate her authority to two assistant matrons and be correspondingly relieved of much routine work, while there are no less than nine other nursing posts of an administrative character which carry a salary of £150, rising by yearly increment of £25 to £250. A group of sisters at the head of their respective departments will enjoy a commencing salary of £140, rising to £200, while another group commence at £110 and rise to £150. Ward sisters are to receive £90 to £130. In all the alterations proposed entail an additional yearly cost of £10.292 in the first year and will eventually cost over £15,000. The proposals were accepted by the Board. The nursing service of the greatest hospital in the kingdom was at stake.

